# Judgments of a Product’s Quality and Perceptions of User Experience Can Be Mediated by Brief Messaging That Matches the Person’s Pre-existing Attitudes

**DOI:** 10.3389/fpsyg.2020.01261

**Published:** 2020-06-09

**Authors:** Ian Walker, Gregory O. Thomas, Sukumar Natarajan, Nigel Holt

**Affiliations:** ^1^Department of Psychology, University of Bath, Bath, United Kingdom; ^2^Welsh School of Architecture, Cardiff University, Cardiff, United Kingdom; ^3^Department of Architecture and Civil Engineering, University of Bath, Bath, United Kingdom; ^4^Department of Psychology, Aberystwyth University, Aberystwyth, United Kingdom

**Keywords:** spillover, halo effect, self-activation, environmental, sustainable, framing

## Abstract

The mediation of an attitude to a product following a brief message is investigated. Statements indicating whether a computer was running on energy from renewable or more conventional sources were presented and users’ experiences were measured. Participants’ pre-existing environmental concern and the satisfaction they expressed with the computers were related, but only when the “renewable energy” message was presented. We conclude that enduring attitudes to environmental concern and situation-specific knowledge can interact in evaluations of a situation – a finding with implications for behavior-change strategies. Theoretically, results are discussed in terms of “spillover” from one behavior to another, the Halo Effect and self-activation, where those with a self-identity of being environmentally conscious have this identity activated by messaging congruent with their self-identity, resulting in an influence of their opinion of a product. Conversely, those with an anti-environmental worldview might rate products more negatively when the product’s environmental credentials are mentioned, presumably because these credentials are not congruent with self-identity.

## Introduction

Many things influence whether a person chooses to buy a product. Brand loyalty, price and usability, review quality, and word of mouth may all be influential. Once the person has invested in the product their experience will be influential in determining whether they continue to use it. It is well-known that the information provided before during or after an event can influence our perception, our memory and our lasting experience of the event itself (Dooling and Lachman, 1971; Bransford and Johnson, 1972; Kozminsky, 1977; Pichert and Anderson, 1977). This is known as “framing.” How the “frames” are couched is also influential in mitigating behavior. For instance, “gain” framing – “*Charities are really helpful in alleviating suffering” –* has been shown by some to be more influential than “loss” frames – “*If we don’t give to charities people will suffer*” (c.f. Morton et al., 2011). However, meta-analyses show that the strength and direction of the effect of each type of framing is unclear and inconsistent (O’Keefe and Nan, 2012) – suggesting that there is no consistent benefit of one type of framing message over the other, even though framing as a whole plays a role. This is a frustration for practitioners who would like to use message framing to influence environmental choices. It is likely that existing attitudes, opinions or similar concepts influence our choices and so provide a key reason why information provision does not work consistently.

We therefore need to consider psychological and situational factors that might explain different responses to a given message, across and within people. Oliver (1980, 1997) proposed the “expectancy disconfirmation model” which predicts that expectancy confirmation results from a behavior that is in line with how we “expect” ourselves to behave, producing a positive feeling (c.f. Boulding et al., 1993). The converse is also true, that behavior that is not in line with an expected attitude (disconfirmation) results in a negative feeling. These positive or negative experiences influence the likelihood of behavior being carried out. Ertz and Sarigöllü (2019) took this further. They identified that a feeling of “satisfaction” might arise following a behavior, partly as a function of “expectancy conformation,” and that the level of satisfaction we feel about a past behavior influences our attitude to other, related behaviors. This suggests that to encourage future behaviors we must work to increase peoples’ satisfaction with related, current behaviors – perhaps through incentivizing the behavior through encouragement and reward.

In our study we focus directly on pre-existing attitudes and how they may influence opinions when “activated” and potentially strengthened with a framing message. Of particular interest here is the psychological concept of “self-activation” – a development of psychological work on self-identity, or the image that we each hold of ourselves (Verplanken et al., 2008; Thomas et al., 2016). As the self is a consistent and enduring construct that is potentially available during every decision, it is plausible that it will be a key variable at play when people evaluate any given message. More specifically, if a message about a product is couched in such a way that it *activates* specific self-concepts in the decision-maker, it is particularly likely that self-identity will play a role in that product’s evaluation.

Verplanken and Holland (2002) found that framing with environmental messaging triggered preferences for sustainable consumer behavior only in those who had previously expressed strong environmentalist values. Thomas et al. (2016) found a more complex relationship between attitudes and opinions, noting that a change in circumstances might be a trigger to self-activation. For instance, moving house may provide a “trigger” to engage with longer-term beliefs about the environment that had not surfaced due to habitual behaviors. Moving house provided the break in the habit which allowed for self-activation, and so a change in attitudes and more environmentally sustainable behavior (see also Verplanken et al., 2008). This all raises the question of whether messages intended to trigger self-concept constructs might specifically be used as a way of influencing decision-making. Here we look at this question in the environmental domain, but there is no reason it might not work in other domains too if messaging and self-concepts, similarly, align.

We already know that straightforward framing messages can have an influence on environmental decisions. Viscusi and Zeckhauser (2006) found that inserting a passage of text explaining climate change at the top of a questionnaire led to participants giving higher estimates of the extent of future global warming. Similarly, Joireman et al. (2010) found that the extent to which people agreed that the human contribution to climate change was a serious issue could be influenced by recent experiences such as exposure to heat-related words (e.g., sunny, burn, sweat). Related to this, Durfee (2006) presented participants with passages to read, and measured readers’ assessments of how dangerous air pollution was to their health. Couching the story in terms that made pollution seem outside anybody’s control led to lower perceptions of risk than if the story discussed the behaviors that had caused the pollution. Critically, none of these previous studies asked whether responses to messages would vary as a function of people’s self-constructs or beliefs. Knowing how the message might differentially activate – and thereby be processed in light of – recipients’ prior self-perceptions would be useful for policy-makers, and would also be theoretically interesting. It is this knowledge gap that we attempt to fill here.

If self-construct activation is a potential mediator between environmental messaging and response, we ought to see that messages congruent with a person’s self-identity will have a global, and not just a specific, effect on their judgments. In other words, if a message about a product produces an effect that goes beyond the narrow evaluation of that product to the core of that person’s self-identity, then the message might have activated a global construct that plays a broad role in the evaluative process.

This all produces an interesting parallel with the so-called Halo Effect – a cognitive bias in the way that we perceive other people (Nisbett and Wilson, 1977). For instance, Dion et al. (1972) found that attractive people were rated as more successful than unattractive people although no information regarding “success” was made available to those making the judgments. The effect is a judgment discrepancy where by the judgment of something nebulous, or not immediately obvious about a person (successfulness), is based on something more “solid” (their appearance). The idea of a Halo Effect has previously been extended from people to products. For instance, Park et al. (2011) indicated that perception of retail brands can influence the perception of a retailer’s own-brand products, which are evaluated more positively if they are sold in a store whose brand is strong. Sörqvist et al. (2015) showed that products labeled as environmentally friendly were rated as tasting better. Elsewhere the “green” halo effect has been influential in judgments of restaurants that source their food locally (Bacig and Young, 2019) and in judgments of wine stoppered with traditional cork closures rather than screw caps (Reynolds et al., 2018). There is also evidence that perceptions of electric cars benefit from the halo effect they enjoy from their being viewed as more sustainable than those that burn fossil fuels.

A “negative halo” is also possible wherein an initial attitude about someone can result in a more general negative opinion of them. Also known as the “devil” or “horns” effect, a single negative judgment of an attribute may influence subsequent judgments of perhaps unrelated dimensions negatively (Forgas and Laham, 2017). For instance, it may be that a judgment of a car as “uncomfortable” may negatively influence a person’s judgment of its efficiency – two unrelated attributes, but the negative judgment of one influences the judgment of the other negatively.

The nature of these positive and negative halo effects is that judgments about a product become globally raised, or lowered, in a way that would fit the idea that whatever specific decision is being made gets influenced by some common underlying process. Given the reasoning explained above, self-activation is a likely mechanism for at least some of this. In the study reported here we take a closer look at these issues and ask whether pre-existing attitudes and self-identity constructs can mediate the effects of a short framing message on attitudes to a product. We looked at the framing information in a message, and how this might bias attitudes to the performance of new computers introduced in a facility at a university. We deliberately did not ask questions about perception of the sustainable nature of the machines. In doing so we avoided any influence of social desirability on judgments that that may be perceived as having political or normative connotations. We instead asked participants only to judge the performance of the machines in the new facility, compared with others they will have used on campus. As such, any increase in positive responses following a framing message is likely to reflect a change in some central mechanism that then influences multiple facets of product evaluation.

## Materials and Methods

Attitudes to a new computer facility at a UK university were measured. Participants completed a questionnaire which recorded demographic as well as attitude and self-identity information. Two versions of the instructions at the start of the questionnaire were used, the subtle difference between them forming the critical independent variable, details of which can be found below.

### Background and Design

The study took place in a new computer laboratory. The computers in this room were part of a technology trial and had been modified so that they ran off Direct Current (DC) energy. This meant that it would be possible, if desired, to run these computers from renewable energy sources like wind or solar power (although, during the study, they drew their power from the national electricity supply which was primarily generated from fossil and nuclear sources at that time). All the computers in the test laboratory were new, and so were faster, cleaner, physically smaller, and potentially more pleasant to use than the other computers on campus. As such, it was important to include a control condition to assess the extent to which responses were affected by the newness of the computers. A second computer suite, of comparable size and layout in the same building, was used to gather control data. The questionnaire used here was the same as for the “conventional sources” condition in the DC suite.

### Participants

Data were collected from 165 people – 125 undergraduate students (75.8%), 20 graduate students (12.1%), one staff member (0.6%) at the University of Bath (19 respondents, 11.5%, did not answer the question about their position, although based on their ages it is probable that all were undergraduates). There were 76 male respondents (46.1%), 83 (50.3%) female respondents, and 6 (3.6%) declined to give their gender. The age range was 18–51 years (mean = 21.3 years, *SD* = 3.84).

### Questionnaire

For the control group, working on standard university computers, and for the participants in the new computer room who were allocated to the “*conventional*” power condition, the text at the top of the questionnaire read as follows:

This short survey looks at how you feel about the library computer you are currently using. This computer gets its power from ordinary mains electricity, mostly generated from fossil fuels and nuclear energy. This questionnaire is completely anonymous, and you are free not to take part, to skip questions you don’t want to answer, or to stop taking part at any time.

For participants working in the new 50-person capacity computer room (known locally as the “DC” room) who were allocated to the “*renewable*” condition, the explanation was exactly the same except that the second sentence was changed to:

This computer has been modified so that it can be run on renewable energy sources.

Below this text were five questions about the experience of using these computers, each rated on a 7-point Likert scale with end-point anchors as described in brackets:

(1) Compared to the other computers on campus, this one is
  [the worst I’ve used] – [the best I’ve used]
(2) Compared to the other computers on campus, this one is
  [very slow] – [very fast]
(3) When looking for a campus computer in the future, would this room be your first choice?
  [definitely would not] – [definitely would]
(4) Would you recommend the computers in this room to your friends or fellow students?
  [definitely would not] – [definitely would]
(5) Does the level of background noise in the room affect your choice of computer room?
  [definitely does not] – [definitely does]^1^

Then followed questions concerned with basic demographic information: age, gender and role (undergraduate, taught postgraduate, research postgraduate, staff). Question 6 was a measure of environmental identity, rated on a 7-point Likert scale

(6) I am the type of person who cares about the environment [strongly disagree] – [strongly agree]

Next, seven items from the New Environmental Paradigm scale of environmental concern (Dunlap et al., 2000) that had previously been employed to measure environmental concern in a similar population were presented (Verplanken et al., 2008). That study found these items had good internal consistency with a Chronbach alpha of 0.84. Each question was assessed with a 7 point Likert scale, with “Disagree” and “Agree” as end points.

(1) We are approaching the limit of the number of people the Earth can support.
(2) Humans have the right to modify the natural environment to suit their needs.
(3) When humans interfere with nature it often produces disastrous consequences.
(4) Human ingenuity will ensure that we do NOT make the Earth unliveable.
(5) Humans are severely abusing the environment.
(6) The Earth has plenty of natural resources if we just learn how to develop them.
(7) Plants and animals have just as much rights as humans to exist.

### Procedure

Data collectors operated outside the two library computer rooms at various times of day. The collectors approached people as they entered each room and offered them a questionnaire. The key manipulation was that participants in the DC computer room received either a “conventional” or a “renewable” version of the survey at random. The survey forms had previously been sorted into random order, based on numbers generated by the website random.org. Each person in the DC computer room who took part in the study received the next form from the pile. The DC computer room was strictly enforced by the university as a silent study area, which meant participants could not discuss the surveys with one another and so discover the experimental manipulation. Users of the control room all received the same, “conventional,” form of the questionnaire. Sixty-three people returned the “renewable” form of the questionnaire, 46 the “conventional” form and 56 were in the control group.

## Results

**FIGURE 1:**
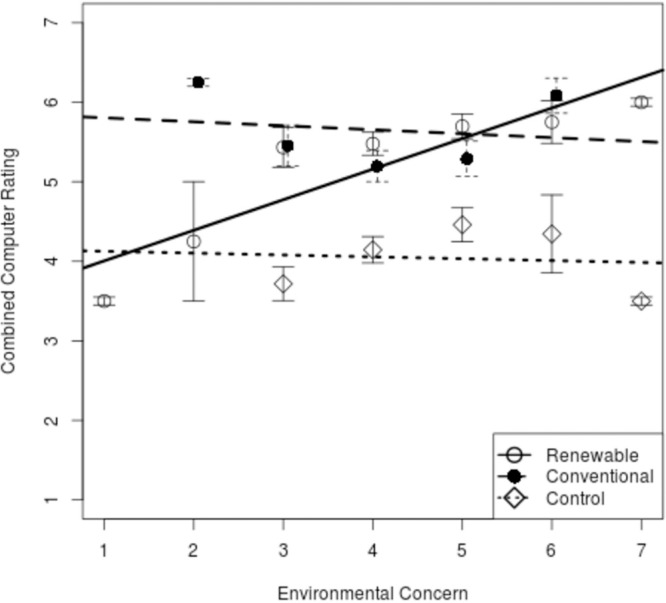
Computer satisfaction ratings (and standard errors of the mean) against environmental concern for the group primed with information about their computers’ ability to use renewable energy, the group primed with information about their computers’ use of conventional energy sources, and the control group that was using older computers.

The four questions that assessed the experience of using the computers had their responses averaged to provide a single computer rating for each participant. This was justified by good agreement between the four measures: Cronbach α = 0.81 (95% CI, estimated from 10,000 bootstrap samples = 0.76–0.85). For each condition, this combined computer rating was compared against environmental concern as measured using the combined New Environmental Paradigm (NEP) scores. For clarity, scores on the NEP were pooled into seven ordered bins (see Figure 1). So that inter-participant variability would not be neglected, standard errors were calculated and plotted. A benefit of reducing the data to a smaller number of points in this way is that it made it more difficult for our regression analyses to reach statistical significance, and so provides a conservative analysis.

Figure 1 shows participants’ computer ratings plotted against environmental concern for each of the three groups. The control group (the lower, near-horizontal line) and the “conventional” group of DC computer users (the upper, near-horizontal line) showed the same slope, but with a higher intercept for the “conventional” group, reflecting the straightforward improvement in user experience achieved by moving from old computers to new computers. The flat lines for these two groups show there is no real relationship between environmental concern and computer ratings.

Importantly, those who received the brief renewable energy message in their questionnaires showed a positive linear relationship between environmental concern and computer ratings – those with a concern about the natural environment rated the computers higher than those who were not concerned, with the unconcerned people giving ratings as low as the control group. Recall that the control group were using substantially older and slower computers than the other two groups. In this condition, users of the much newer computers produced scores in the same range as the control group using the older computers, provided they had very low environmental concern and had been told the computers used renewable sources.

The data were examined using analysis of covariance (ANCOVA), as we were predicting a continuous measure from a combination of continuous and categorical variables. No significant effect of environmental concern on computer ratings was shown *F*(1,11) = 2.40, MSE = 0.23, *p* = 0.150, as might be expected given the essentially flat slopes for two of the three groups. However, there was a significant effect of experimental condition on computer ratings, *F*(2,11) = 18.54, MSE = 0.23, *p* = 0.0003, showing that the intercepts for the three groups were not all the same. There was also a significant interaction between experimental condition and environmental concern, showing that the slopes were not equivalent across the three conditions, *F*(2,11) = 4.50, MSE = 0.23, *p* = 0.037.

As Figure 1 shows, this interaction was caused by the “renewables” group showing the surprisingly different form of relationship compared to the other two groups.

To confirm that the ANCOVA findings were not a product of our decision to group participants into 21 groups (three conditions × seven levels of environmental concern), a regression analysis was performed to predict each person’s computer rating from their raw environmental concern score and two dummy variables. The first dummy variable identified participants in the new computer room who received the renewable message or not; the second identified participant in the new computer room who received the conventional-fuel message (people in the control condition scored zero for both dummy variables, and so acted as the reference group for the analysis, as they were not in either of these conditions). This analysis agreed with the ANCOVA above by showing a significant interaction between environmental concern and the renewable-message dummy variable (*b* = 0.41, *t* = 2.31, *p* = 0.04). There was no significant interaction between environmental concern and the conventional-fuel-message dummy variable (b = 0.03, *t* = 0.12, *p* = 0.90). The significant positive effect of the renewable message × environmental concern interaction coefficient confirms that computer ratings tended to increase with environmental concern, but only when the renewable energy message was also present.

### Mediation of Judgments by Self-Identity

**FIGURE 2:**
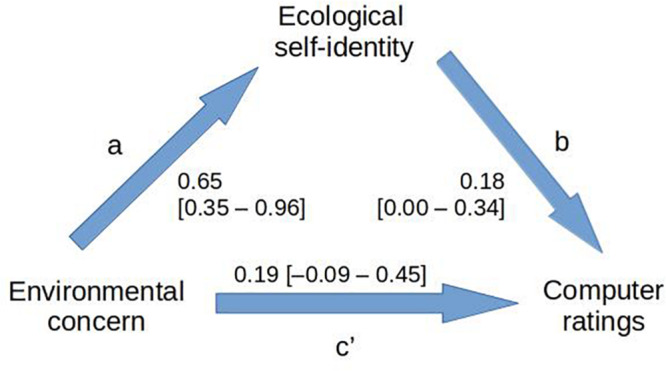
Mediator analysis in which the ability of ecological self-identity to mediate the relationship between environmental concern and computer ratings is assessed. The measure of interest is the product of the unstandardized regression coefficients *a* and *b*, where *b* is calculated whilst controlling for *c’*. Values in brackets are 95% confidence intervals obtained through 10,000 bootstrap samples.

In this section we complete a separate but related analysis. We wished to know whether ecological self-identity might mediate the relationship between environmental concern and computer rating (Figure 2). Ecological identity (“I am the type of person who cares about the environment”) and environmental concern (rated on the NEP scale) were found to be related, but not redundant, concepts, correlating with *r* = 0.35.

We investigated the possible mediating effect of ecological identity on the “environmental concern → computer rating” relationship using the bootstrap mediator procedure described by Preacher and Hayes (2008, pp. 879–880). Bootstrapping is a nonparametric procedure in which the variability of a statistic is estimated by repeatedly sampling (with replacement) from the data that have been collected, calculating the statistic of interest each time, and examining the variability of these statistics across successive samples. If one has a set of *n* scores and repeatedly takes samples of size *n* from these, with replacement, calculating the mean each time, the distribution of these means can provide confidence intervals for the mean of the original scores (In the simplest method, one arranges the estimated means from the lowest to the highest and finds the central 95% of these to estimate the confidence interval).

In this study, random samples of participants were taken from the pool of available data, with replacement, 10,000 times; the linear regression model predicting computer-quality ratings from environmental concern was computed each time, as was the linear model predicting computer ratings from ecological self-identity whilst controlling for environmental concern.

The key measure from this mediator analysis was the product of the unstandardized regression coefficient predicting self-identity from environmental concern and the unstandardized coefficient predicting computer quality rating from self-identity with environmental concern controlled (Shrout and Bolger, 2002; Preacher and Hayes, 2008). Bootstrap analysis with 10,000 replications estimated this value to be 0.12 with a 95% confidence interval of 0.02–0.28. As this interval does not include zero, the mediating effect of ecological self-identity on computer ratings is statistically significant. The results show that predicting computer quality ratings from measures of environmental concern is more accurate when we consider the mediating effect of the participant’s self-identity.

The results are clear – the relationship between environmental concern and the ratings of the product (in this case a computer) are influenced by how the person self-identifies – in this case whether the person regards themselves as “environmentally concerned” or not.

## Discussion

We asked whether a brief message about the sustainability of the power source for a computer might influence the person’s experience of using it. We also looked at whether the person’s self-identity may play a role in their judgment and whether this mediated the influence of the of the message. The judgments made about the product were non-environmental in nature and so not directly related to the framing message. Instead participants were asked to judge the overall quality of the product. What we see then is that for those with a pre-existing pro-environmental identity or attitude, a message that corresponds with that self-identity or attitude can influence the behavior that follows, even when this behavior is in a different (non-environmental) domain. The finding is consistent with the idea that the message activates a pervasive central process (plausibly, the self) that affects a broad range of subsequent decisions.

### Spillover and Self-Identity

“Spillover” is a term used to describe a propensity for an environmentally conscious behavior in one domain to be seen elsewhere in a different domain (Poortinga et al., 2013). In the context of this paper, the “attitude” is the behavior to which the spillover refers. Spillover can be in the same direction as the original behavior (a positive attitude to sustainability spilling over to a positive attitude to the secondary behavior). The spillover may be negative (Thøgersen and Ölander, 2003) where the secondary behavior is in the opposite direction to the initial behavior. Finally, the spillover may be neutral, with no spillover observed at all. Attitude has been discussed in terms of a spillover behavior previously, for instance Asvatourian et al. (2018) showed that a positive attitude toward pro-environmental behaviors may spillover into a, similarly positive attitude toward healthy food, related to lower greenhouse gas emissions, than non-healthy diets. Verfuerth et al. (2019) looked at how spillover might influence responses toward an intervention to influence food choice (reduced meat intake). They assessed identity change in a group of employees before and after the intervention using interviews and a visualization task in which participants physically placed various environmentally relevant comments around an outline drawing of a person – a representation of themselves. Using this novel method, the authors concluded that there was some spillover to the food choice intervention, but only in those for whom the target of the intervention (meat reduction) was relevant to the person’s sense of self-identity. Lauren et al. (2019) considered whether self-perception could mediate intentions to behave in an environmentally sustainable way in the future. Self-perceptions were “activated” with a checklist of behaviors that participants in the high-activation condition were to check if they “sometimes did” or in the low activation condition when they “always did” them. Results showed that those in the high activation group were more likely to indicate that they would take part in sustainable behaviors in the following 6 months than those in the low activation group.

In our study, using a very different method to Verfuerth et al. (2019) and Lauren et al. (2019), we found that immediately tested attitudes toward the computer being used were influenced by a short framing message but that this was only the case for those with a relevant, existing environmental concerns. As such it could be concluded that in this case, non-specified environmental concerns, indicated in the relevant question of our questionnaire, may have “spilled over” to influence the person’s evaluation to the computers, but only if those concerns had been “activated” by the framing message. This in part supports the findings of Verfuerth et al. (2019), and the predicted behavior influence identified by Lauren et al. (2019), with a different approach and using a different experimental example.

Important here is that the judgments made in our procedure did not relate to the “environmental” credentials of the computer being used, only it’s “quality.” As such, the “spillover” was evidenced in a different domain – the deliberately nebulous “quality” of the product rather than it’s “environmental” merits. Our results show that framing messages that match the person’s attitude, and activate their existing opinions, might result in more “global” changes in attitude. Here we have demonstrated a “spillover” of sorts from one category of attitude (environmental concern) to another (opinion of quality unrelated to the environment).

The effect we have seen here is considerable. Prentice and Miller (1992) suggested that, as well as looking at straightforward measures of effect size such as *r*^2^, the magnitude of a psychological effect can also be gauged by how easily it can be invoked in experimental settings: a response triggered by even the smallest intervention is likely to represent an important psychological mechanism. In our study we have identified a reliable effect following a small change to a single sentence in the text of an instruction. As such it follows that the psychological processes at work here are likely to be powerful and pervasive, and it is these psychological processes and the mechanisms involved that we need to address here.

Earlier we refer to behavioral “spillover,” the effect of an intervention on subsequent behaviors not directly targeted by that intervention. In this case, the intervention is the framing message that we describe as resulting in self-activation where it is congruent with the person’s prevailing attitude to the environment, or not in the case where the message and the attitude differ.

Elsewhere in the literature, evidence for spillover is mixed. A consensus seems to be that there is some evidence of pro-environmental activity on one area spilling over to pro-environmental behavior elsewhere (Fanghella et al., 2019). The magnitude of the spillover, however, both in terms of the size of the influence on other behaviors or attitudes, and on the range of behaviors and attitudes influenced, seems to vary. Thomas et al. (2016), for instance, looked at a particular intervention: the introduction of legislation regarding the use of plastic bags in Wales, United Kingdom. They investigated spillover into other environmental behaviors such as turning off taps, and using public transport and concluded that the bag-reuse legislation had a minimal effect on other related pro-environmental behaviors. The results described in this paper can be described in terms of spillover, but a cognitive bias, the Halo Effect, needs also to be considered in the mechanism by which the judgments came about.

Our findings can be thought of in these terms. The results indicate that participants’ behavior regarding the satisfaction they had with the computer was a result of spillover of sorts. We deliberately did not ask for an opinion of the environmental credentials of the computer. Instead the brief message indicating that the computer ran on renewable energy, resulted (in those with a congruent attitude to the environment) in self-activation. We suggest that this in turn resulted in a “halo” effect surrounding the computer being tested. Those who saw it as “green” following the framing message about the origin of the power it used rated its performance higher under conditions of self-activation. In terms of the halo effect, we can say that the nebulous or more abstract contentment with the machine was influenced by the rather less ambiguous information about its environmental credentials in the framing message, but only in those who held congruent environmentally concerned views. The framing message not only activated the person’s self-perception, but placed the computer in a favorable, “green” light which can be described in terms of “green halo” mentioned earlier. Such a “green” halo has been shown elsewhere.

### A Negative “Halo”

In the experiment, if people were told their computers used renewable energy, the ratings of the most environmentally concerned users were comparable to those in the “conventional” power condition who were using the same computers (Figure 1), whereas those who were unconcerned with the environment gave ratings comparable to the users of older, slower computers in the control condition, despite actually using the newer faster machines. As such, we can say that briefly mentioning renewable energy sources to people who were more anti-environmentalist led to the overall experience of using the computers feeling worse, a negative influence, and this negative effect may be of more importance in the data than the positive effect of a framing message on someone who already has environmental concern. An important implication then of the results of this study might be that messages about a product’s “environmental” credentials might actually be counter-productive. Such messages may invoke defensive responses from those who feel negatively toward an environmentalist stance. As such the immediate response that follows the message may not be as hoped, and the “spillover” that follows may actually be negative, consistent with a negative halo, or “horns” effect.

### Wider Relevance

If information about the environment has the sort of global effects we have suggested on those people who are untouched, or actively unimpressed, by environmentalists’ messages, then this is likely to be important at the population level. Whereas it is clear that most people in this UK university-based sample fell into the upper half of the environmental concern scale, there are many in the wider population who would score lower. For example, an opinion poll from the US found that roughly half of Americans do not take climate change seriously (Brenan and Saad, 2018). Bergquist and Warshaw (2019) aggregated data for 170 polls, and concluded that although there is evidence that although continued temperature rises will, they anticipate, cause concern for the environment to grow in general, the warming alone will not generate a consensus in the public about it. We suggest that this may be because messaging about the environment may well have very different results as a function of the views held by those receiving the messages and the proportion in the US alone who do not consider global warming as a significant issue, the effect of the messaging could be counterproductive if not very carefully targeted. There are significant implications of these data for policy makers for whom legislation may be designed to generate a more sympathetic treatment of the environment. Such legislation and the messaging produced to encourage a desired behavior changes may well have the opposite effect in those for whom the environment is not a concern. Worse, in those for whom such environmental legislation is an indication of liberal ideals that they actively reject, moves to generate climate-sympathetic behaviors might be counter-productive. We suggest that where further research should consider the (negative) halo effect as part of the mechanism in explaining spillover and the effects of self-activation.

## Conclusion

Ecological self-identity, as assessed by the extent to which people saw themselves as “environmentally concerned,” mediated the relationship between environmental concern and ratings of the computers partly as a function of the halo effect the computers enjoyed when labeled as using “renewable” energy. Environmental concern affected computer ratings in part directly and in part through a process related to self-identity. Whilst certainly not definitive evidence, this analysis supports our tentative theory in which mention of the computers’ environmental properties activated people’s self-identities and thus biased subsequent judgments in line with those identities, even when those judgments were outside the environmental domain. If this interpretation is correct, a practical implication is that messages about a product’s “green” credentials can risk being counter-productive. As such messages might invoke defensive responses from those who feel negatively toward climate change messages, rather than positive responses in those concerned with climate change, the effect could be to make the product look poor to those who have anti-environmentalist attitudes without any concomitant improvement in the experience amongst those who are ecologically concerned. Particular resentment against environmental legislation can be predicted in those who identify themselves as anti-climate-change, important to note when developing relevant policy.

## Data Availability Statement

The datasets generated for this study are available on request to the corresponding author.

## Ethics Statement

The studies involving human participants were reviewed and approved by University of Bath Psychology Ethics Committee. Written informed consent for participation was not required for this study in accordance with the national legislation and the institutional requirements.

## Author Contributions

IW, GT, and SN: original conception, planning of the experiment, data collection, and analysis. GT, IW, SN, and NH: theory development and initial manuscript construction. IW and NH: manuscript finalized for frontiers, with additional theoretical development.

## Conflict of Interest

The authors declare that the research was conducted in the absence of any commercial or financial relationships that could be construed as a potential conflict of interest.

## Footnotes

1 This last question was included at the request of facilities organizers and was not included in the analysis.
